# Spousal Violence in Bangladesh: A Call for a Public-health Response

**Published:** 2008-09

**Authors:** Heidi Bart Johnston, Ruchira Tabassum Naved

**Affiliations:** ICDDR, B, Mohakhali, Dhaka 1212, Bangladesh

**Keywords:** Gender, Review literature, Violence, Violence, Spousal, Bangladesh

## Abstract

Spousal violence against women is a serious public-health issue. Although there is a growing body of literature on this subject, there are still many unanswered questions regarding the prevalence of this violence, the risk factors, the consequences, and how to address the issue. The purpose of this literature review is to organize and synthesize the empirical evidence on spousal violence against women in Bangladesh and to provide direction for both researchers and practitioners for future work in this area. The review suggests that spousal violence against women is high in Bangladesh. The list of correlates is long and inconclusive. Although there is evidence on adverse consequences of this violence on health of women and their children, more research is needed to explore the multifaceted consequences of violence for women, children, families, and communities. Action research is needed to develop and test preventive and curative interventions.

## INTRODUCTION

Over the past 15 years, the growing body of research on violence against women has brought the issue from a position of near-invisibility to being recognized as having far-reaching health and economic impacts for women and societies (1). In recent years in Bangladesh, researchers have documented the prevalence of physical, sexual and emotional abuse, associations between violence and physical and mental health, characteristics associated with the risk of experiencing violence, particularly spousal violence, and attitudes of women towards spousal violence and coping strategies.

The Government of Bangladesh has made strides in harmonizing national legislation with international commitments to protecting the rights of women and eliminating violence against women. For example, Article 27 and 28 of the Constitution specifically guarantee equal rights of women before the law. Other acts to protect the rights of women include: The Dowry Prohibition Act (1980; 1986), The Prevention of Women and Child Repression Act (2000), and The Cruelty to Women Ordinance (1983). Furthermore, Bangladesh has ratified the Convention on the Elimination of All Forms of Discrimination against Women (1979), which calls for the protection of women from gender-based abuse and negligence. The Government is committed to eliminating violence against women by 2015 in Bangladesh, as stated by the Minister of Health and Family Welfare and as detailed in Target 6 of Millennium Development Goal 5 to improve maternal health [presentation at the High Level Forum on Health MDGs in Asia and the Pacific, Tokyo, 2005]. Although domestic violence, particularly violence perpetrated by husbands, is the most common violence against women in Bangladesh (2), the laws mentioned above do not address domestic violence that do not result in injury. Moreover, enforcing and implementing the existing laws, acts, and agreements even for severe violence have proven to be challenging.

In this paper, we summarize the available research findings to document what is known and what needs to be known to reduce the incidence of spousal violence effectively and efficiently in Bangladesh. We reviewed available evidence on spousal violence in Bangladesh and, where appropriate, drew from related topics or other geographical areas. Information in the paper comes from a review of publications and other reports on spousal violence in Bangladesh and from other countries as appropriate; a review of publications and other reports on deaths from intentional injury (suicide and homicide) among women of reproductive age in Bangladesh; and original analysis of ICDDR, B surveillance data on deaths from intentional injury. We included data on deaths from intentional injury since spousal violence can result in either suicide or homicide; furthermore, intimate partner homicides may be labelled as suicides, and actual suicides may be an indicator of mental instability resulting from violence and other causes (3). Data on deaths from intentional injury are collected in accessible surveillance datasets, whereas data on spousal violence are not. Following the World Health Organization (WHO) definition of intimate partner violence, in this paper, spousal violence refers to physical, sexual, or emotional abuse perpetrated by an intimate male partner, or husband, against a woman, or wife (4).

## REVIEW OF EVIDENCE

The growing body of research suggests that spousal violence is highly prevalent in Bangladesh. In 2001, about 60% (59% in an urban area and 60% in a rural area) of women reported having ever-experienced physical or sexual spousal violence. Consistent with the global trend, the overwhelming majority of physical and sexual violence against women was perpetrated by husbands, not by other persons (4). While disturbingly high, the rates of spousal violence documented in Bangladesh are not unusual. A recent study from the USA reported that nearly half (44%) of more than 3,400 female members of a Seattle-based health cooperative reported having experienced spousal violence during their adult lifetime (2).

### Physical violence by husbands

Several studies have measured rates of spousal physical violence in rural Bangladesh in the decade of 1992–2002. In 1992, Schuler *et al*. found that 47% of women of lower socioeconomic status in six villages in three districts of Bangladesh reported ever having experienced physical domestic violence (5). Nineteen percent of women reported having been beaten in the past year. In 1993, 42% of women in Jessore and Sirajganj reported being beaten by members of their households in the recent, but unspecified, past (6). Forty-three percent of women in a Gazipur sample reported physical violence in the past year (7). In 2000, spousal violence was reported in 51% of households of a rural conservative area of Bangladesh (8). In 2001, 42% of women in a rural area reported being physically assaulted by their husbands at some point in the past; 16% reported this over the previous year (4). In 2002, 67% of women in the same six villages as the 1992 estimates reported having ever been beaten by their husbands, and 35% reported having been beaten by their husbands in the past year (9).

While the numerous studies demonstrated clearly that physical spousal violence is widespread, methodological variations prevent identification of time trends. These methodological variations include: different definitions of physical violence; different reference periods; differences in perpetrators; cultural and geographic variations; socioeconomic variations; and differences in primary focus of data collection. In several studies, respondents defined physical violence for themselves, leaving room for variation at the individual level (5-7). Bates *et al.* and Schuler *et al.* looked at violence among lower socioeconomic groups, which tend to have higher rates of violence (9,10). While these two studies look at violence in the same populations, respondents defined violence in the first study, but in the second study, the investigators used the WHO definition of violence against women and used a series of behaviourally-explicit questions.

Physical violence can be severe and recurring. Almost one-fifth (19%) of rural and urban Bangladeshi women who experienced physical violence within the past year reported that the violence was severe, including having been hit with a fist, dragged, kicked, or threatened with a weapon (11). Eighty-nine percent of urban women and 86% of rural women who had ever experienced physical violence reported that the physical violence occurred more than once or many times (4), indicating that these acts of violence are not isolated, but part of a pattern of continuing abuse. Surveillance data suggest that nearly 10% of deaths of women of reproductive age in Matlab from 1982 to 1998 were directly attributable to violence from husbands and in-laws (12).

Available data indicate strong associations between violence by intimate partners and physical health of women. While similar to other countries, most injuries reported to a large 2001 WHO study on spousal violence in rural Bangladesh were minor (bruises, abrasions, cuts, and bites), and the majority of ever-injured women reported losing consciousness due to a violent incident, which was quite uncommon for other countries covered by the WHO study. Eighty percent of rural women who reported ever having been injured by their husbands reported having needed healthcare for an injury (4) (Table).

**Table.** Severity and frequency of injuries among women ever injured by an intimate partner in urban and rural Bangladesh

Rural/urban	Women ever physically abused by husbands		Women ever injured						Ever needed healthcare for injuries (%)
	Ever injured		Frequency of injury			If ever unconscious			
	No.	%	1-2 time(s) (%)	3-5 times (%)	>5 times (%)	<1 hour (%)	>1 hour (%)	Never (%)	
Urban	146	27	58	25	16	35	15	50	69
Rural	138	25	55	33	12	29	29	42	80

Source of data: Garcia-Moreno *et al*., 2005:58 (4)

Spousal violence can result in death, but as the social stigma associated with homicide are strong and official penalties are very severe, deaths attributable to and associated with domestic violence are difficult to enumerate even in surveillance systems. In a study of deaths from intentional injury to women aged 15-44 years registered in the ICDDR, B's Health and Demographic Surveillance System in Matlab from 1992 to 1998, verbal autopsy data linked violence by husbands and/or other relatives to 10% of homicides. The investigators suggested that this is probably an underestimate of spousal violence-related homicide (12).

### Sexual violence by husbands

While physical violence is well-documented in Bangladesh, a very few studies have investigated sexual violence. The WHO study of 2001 found that 37% of women in the urban study site and 50% of women in the rural study site reported having been sexually violated by their husbands at some point in the past, and 20% of urban and 24% of rural Bangladeshi women reported having been sexually violated by their husbands in the previous year (4). There is a significant overlap between women who have experienced physical violence and sexual violence: 48% of rural women and 41% of urban women who ever-experienced partner violence reported having experienced both (4).

No legislation criminalizes sexual violence within marriage in Bangladesh, and forced sex, or rape, within marriage is prevalent, particularly among the very young couples. Twenty-four percent of urban women and 30% of rural women reported that their first sexual experience was forced. Women aged less than 15 years at the time of first sexual encounter were more than twice as likely to have forced first sex compared to women who were aged over 18 years at first sexual encounter (4).

### Emotional abuse by husbands

Physical and sexual violence are more readily quantifiable than emotional abuse. However, results of qualitative research demonstrated that emotionally-abusive acts might be more devastating than physically-abusive acts (4). The issue of emotional abuse is complex, and data are scarce. In Bangladesh, 44% of urban women and 31% of rural women in 2001 reported ever having experienced emotional abuse—defined as those including insults, humiliation, intimidation, and threats—and 29% of urban women and 20% of rural women reported experiencing emotional abuse in the past year (4). Data collected in Jessore and Sirajganj, in 1993, showed higher rates of verbal emotional abuse, with 79% of currently-married women in one rural area and 89% in another rural area reporting ever having experienced verbal abuse (13). Again, these differences in levels of abuse might be attributable to methodological differences and sociocultural variations among the study sites.

### Association between spousal violence and physical and mental health

Spousal violence yields not only the direct health impacts of injury and mortality, but also contribu-tes to the burden of disease by indirectly impacting other health outcomes (4). In Bangladesh, women with a lifetime experience of physical or sexual violence, or both, by their husbands were significantly more likely to report poor or very poor health (adjusted odds ratio [AOR] 1.7; 95% confidence interval [CI] 1.2-2.2 for urban women and AOR 1.4; 95% CI 1.0-1.8 for rural women) and to report problems with walking or carrying out daily activities, pain, memory loss, dizziness, and vaginal discharge (4).

Abused women have a diminished ability to care for themselves and their children. Results of one analysis showed that consumption of food supplements provided by the National Nutrition Programme was less for pregnant women who experienced physical violence during pregnancy (11). Results of another study showed that physical abuse of a mother was associated with lower quality of maternal-infant feeding interaction, overtly negative maternal behaviours, and lack of interaction from the infant's side (14). Women with more than two years of education had an increased risk of under-five deaths of their female offspring if ever exposed to severe physical violence (adjusted hazard ratio 2.2, 95% CI 1.06-4.50) or to a high level of controlling behaviour in marriage (adjusted hazard ratio 2.5, 95% CI 1.30-4.90) (15).

Women who had experienced spousal violence were 3.5-4 times more likely to have thought of ending their lives and six times more likely to have attempted on one or more occasion(s) to end their lives compared to women who had not experienced spousal violence (4). Both physical and psychological spousal violence contributed to contemplation of suicide by women (3). In an analysis of actual suicidal deaths among women aged 15-44 years in Matlab, 46% were preceded by quarrels and/or serious tensions with husbands (12). Sexual violence, however, has not been associated with suicidal ideation in Bangladesh (3).

**Fig. 1:**
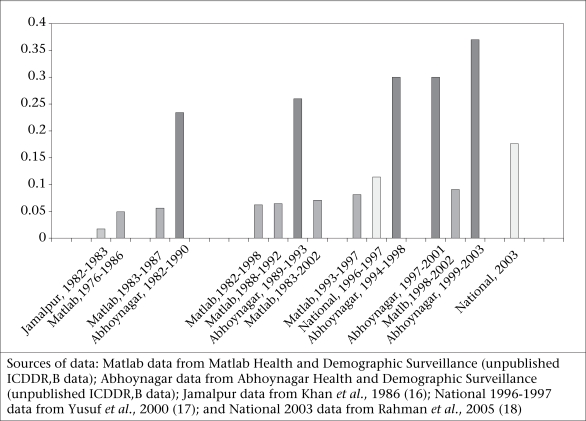
Suicide as a proportion of deaths of women aged 15-44 years, 1976–2003

Suicide is an issue of growing concern in Bangladesh with some areas reporting 37% of all deaths of women aged 15-44 years attributable to suicide. Longitudinal data suggest that the percentage of deaths attributable to suicide among women, particularly young women of reproductive age, is increasing (Fig. 1).

**Fig. 2:**
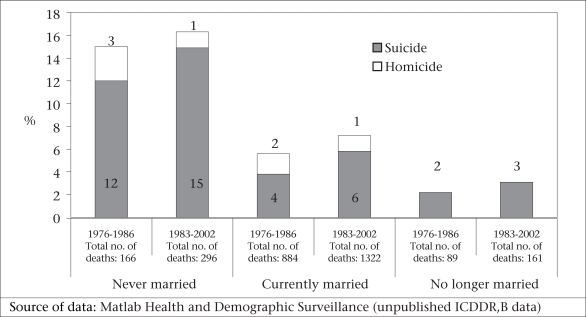
Percentage of deaths of women aged 15-49 years attributable to suicide and homicide in Matlab, 1976–1986 and 1983–2002

**Fig. 3:**
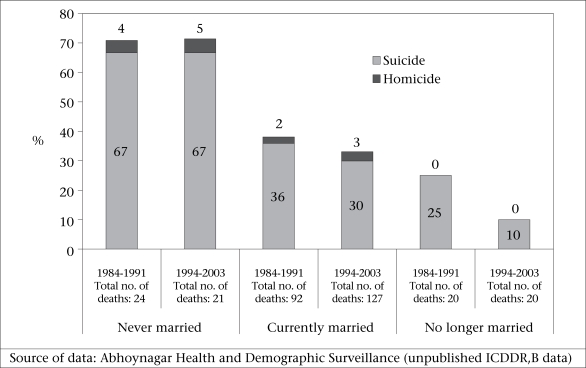
Percentage of deaths from intentional injuries (see text) and total numbers of deaths in women aged 15-44 years by marital status, Abhoynagar, 1984–1991 and 1994–2003

Never-married women are more likely to end their lives in suicide than other marital categories (Fig. 2). In Matlab, during 1983–2002, 15% of deaths among never-married women aged 15-49 years were attributed to suicide (Matlab Health and Demographic Surveillance System—unpublished data). In Abhoynagar, during 1994–2003, 67% of deaths among never-married women aged 15-49 years were attributed to suicide (Fig. 3) (Abhoynagar Health and Demographic Surveillance System—unpublished data). As these suicides take place prior to marriage, they are not directly related to violence from a husband and, in Bangladesh, could not technically be defined as related to spousal violence. However, the association between extramarital pregnancy and suicide suggests that suicides among unmarried women of reproductive age may be linked to extramarital sex—forced or consensual—and subsequent unwanted pregnancies (19).

**Fig. 4:**
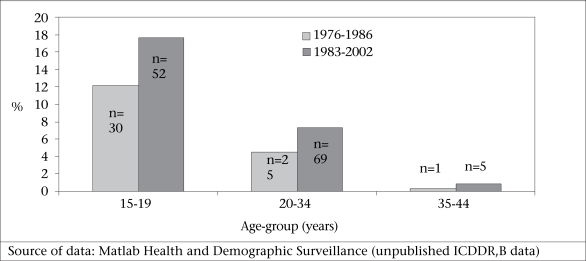
Percentage of deaths attributable to suicide by age-group in Matlab 1976–1986 and 1983–2002

**Fig. 5:**
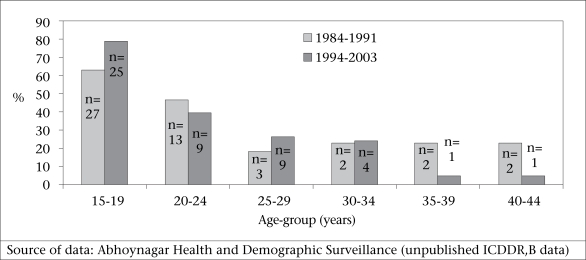
Percentage of deaths attributable to suicide by age-group in Abhoynagar, 1984–1991 and 1994–2003

Suicides among very young women of reproductive age are extremely high in Matlab, where, during 1994–2003, 78% of deaths of women aged 15-19 years were attributable to suicide (Fig. 4). In Matlab, during 1983–2002, 18% of deaths of women aged 15-19 years were attributable to suicide (Fig. 5). These data also suggest that, among this age-group, suicidal deaths are increasing (Fig. 4 and 5).

### Correlates with spousal physical violence

Analyses of correlates with spousal violence have shown associations between spousal violence and age, education, wealth, and other factors. This section presents findings for possible correlates with spousal violence from six studies. There is not an absolute agreement across these studies, perhaps because of variations in the study samples, the geographic areas covered, and the methodologies. Despite the variability, the evidence identifies important relationships that will help guide future prevention efforts.

Youth is a risk factor for violence within marriage (5,6,12,20). In Bangladesh, many marriages are arranged for girls when they are in their teens (18). The status of young wives is usually low. Consistent with their low status, younger women in Bangladesh are more likely than older women to be abused (5,6,9,11).

**Fig. 6:**
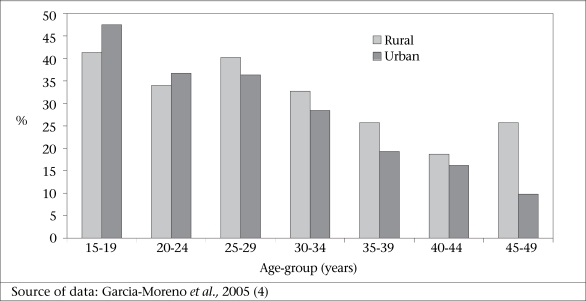
Percentage of women aged 15-49 years experiencing physical or sexual violence by husbands in the last year by age-group and by urban or rural areas in Bangladesh

However, as with other variables, there are variations by area. In urban Bangladesh, in 2001, 48% of 15-19 years old women reported sexual or physical violence from their husbands over the past year compared to 10% of women aged 45-49 years, indicating a significant negative relationship. However, the difference in the proportion of women reporting violence in these extreme reproductive age-groups is less pronounced in rural areas. Responding to the same survey also in 2001, 41% of women aged 15-19 years from a rural area of Bangladesh reported current violence compared to 26% of women aged 45-49 years (4,11), and this relationship was not significant (Fig. 6). Koenig *et al* found a significant negative relationship between age and violence in Jessore but an insignificant difference in Sirajganj, a relatively more conservative area (6).

Age of husbands was not associated with violence according to two quantitative studies from the late 1990s (12,20). However, based on qualitative data from a conservative rural area, Bhuiya *et al* inferred that husbands aged less than 30 years were substantially more likely to beat their wives than husbands aged over 50 years (8).

Pregnancy may have a protective effect from spousal and intimate partner violence in Bangladesh, as in many countries (4,21). Ten percent of ever-pregnant women in an urban site and 12% in a rural site reported being beaten during a pregnancy. Still, the abuse is unsettling: of women abused during pregnancy, 37% of urban women and 25% of rural women reported being punched or kicked in the abdomen. In 99% of cases in both urban and rural areas, it was the biological father who beat the woman during the pregnancy (4).

Violence directed at pregnant women can be extremely severe (4,22,23). One study, using data from a rural area of Bangladesh, compared deaths from intentional injuries (homicide and suicide) among pregnant or recently pregnant women with women who were not pregnant. Pregnant women (including those who had recently delivered within 90 days) aged 15-19 years were 2.6 times more likely to die from intentional injuries than women of the same age who were not pregnant or had not recently had a baby, and much more at risk than older exposed women (23). Results of the study suggest that severe stress are associated with pregnancy among very young women of reproductive age. These findings need to be explored further. A subsequent study might investigate different levels of death by intentional injuries among pregnant women by marital status.

The relationship between socioeconomic status and spousal violence in Bangladesh is undetermined. Results of several studies showed a strong inverse relationship between spousal violence and socioeconomic status (6,9,12). However, no significant relationship was found between spousal violence and level of income in the rural or urban study site when Naved and Persson controlled for factors, such as education and intergenerational transmission of violence (11).

Results of several studies conducted recently in Bangladesh showed a decrease in spousal violence as levels of education increase (6,9,11). Bates *et al* found that women with six years of education or more had significantly reduced odds of experiencing spousal violence (9). Koenig *et al* found that even some level of primary school attendance is highly statistically significant and inversely related to the risk of spousal violence (6).

For men in rural Bangladesh, having less than six years of education did not have an impact on perpetrating spousal violence (6,11,12). In an urban area and in some rural areas, 6-11 years of education did have an inverse relationship with men perpetrating spousal violence (6,11). Results of one study showed that, in a rural area, less than 11 years of education had no diminishing impact on the risk of perpetrating spousal violence, but educational levels of 11 years and higher did have an inverse relationship with perpetrating spousal violence. The authors suggest that a higher level of male education may be needed in rural areas to outweigh the influence of conventional gender roles (11).

Analyses of the impact of family characteristics on the risk of spousal violence have included: registration of marriage, existence of a dowry agreement at marriage, reliance on wife's natal family for support in crises, level of spousal communication, structure of the family (extended or nuclear), the number and sex of children, whether or not the mother of the wife was abused by her father, whether or not the mother of the husband was abused by his father, membership of wife in savings and/or microcredit groups, and income generation of women.

The strongest factor associated with violence of husbands towards their wives in both urban and rural areas was a history of abuse of the husband's mother by his father, according to analysis of Naved and Persson (11). The relationship between family structure and domestic violence is also statistically significant; interestingly, residing in an extended family had a protective effect against the risk of spousal violence compared to living in a nuclear family (6). Within marriage, a high level of spousal communication also had a protective effect, a finding consistent with the idea that men resort to physical violence when they do not have positive channels of communication with their wives (11).

While dowry has been illegal in Bangladesh since 1980, the practice persists, as does the perception that a generous dowry will strengthen the position of woman within her marriage (9). Dowry demands have been found to be positively related to the risk of spousal violence in both urban and rural areas (9,24-26). A review of newspaper articles in Bangladesh linked dowry-related violence to homicide: in articles published from 1983 to 1985, 22% of 229 reported deaths among rural women were related to dowry as were 10% of 41 deaths among urban women (27). Naved and Persson argue that it is not dowry *per se* but the patriarchal attitude of the families demanding the dowry that is positively associated with physical violence against women (25).

A reliance on wife's natal family for support was not related to spousal physical violence (11). While results of several studies showed that the number and sex of children was unrelated to spousal violence, Schuler identified a significant inverse relationship (5,6,12).

International evidence indicates that participation in female workgroups can lower the risk of exposure to intimate partner and spousal violence (28). Bangladesh is well-known for engagement of women in group-based microsavings and credit programmes, which in the mid-1990s engaged more than 3.5 million participants in Bangladesh, many of whom were women (29). The garment industry, another sector with largely female workforce in Bangladesh, engaged 1.5 million workers in 1997–1998, 90% of whom were women (30). The numbers are likely to be much higher today.

Multiple studies have investigated the relationship between spousal violence and income-generating activities of women in Bangladesh; results of studies are, however, inconsistent and inconclusive. Several studies have shown a negative relationship between spousal violence and membership in microcredit groups, whereas others showed a positive relationship, and still others showed no significant relationship (6,9-11). The same range of relationships exist between spousal violence and other income-generating activities of women (6,9-11). This inconsistency may be explained by differences in the local acceptability of women's engagement with microcredit groups. The leading explanatory hypothesis is that the stresses associated with changing gender roles put women at an increased risk for spousal violence (10).

In areas where engagement of women with credit groups of non-governmental organizations has become common and gained acceptance; participation in such workgroups may no longer promote violence. The level of spousal violence decreased as the length of association with a microcredit programme increased (6,10). Furthermore, the percentage of women in a community who belonged to savings and credit groups had a significantly negative association with the risk of spousal violence, with the risk of violence diminishing as a higher proportion of women in the community participated in such groups (5,6).

In terms of other income-generating activities of women, Bates *et al* found that, while women believed that having an income would be protective against spousal violence, data from the same population indicated that women with personal earnings experience more domestic violence than women who do not have personal earnings (9). The risk of spousal violence was significantly higher among women who used their own earned income to cover household expenses (9). Naved and Persson noted a similar trend in rural, but not urban areas (11).

Two other factors—migration of male labour and addictive behaviour of the husband (drugs, alcohol, and gambling)—impact on spousal relations and deserve to be taken into account in future quantitative research (26).

### Attitudes of women and coping strategies

Attitudes of women towards spousal violence vary by whether or not they have experienced spousal violence. An association between experience of physical or sexual violence and agreeing that violence is sometimes justified has been identified, but the direction of the association is not known. Sixty-four percent of urban women and 86% of rural women who have ever experienced spousal violence reported that a husband has the right to beat his wife under certain circumstances. These circumstances range from not completing housework adequately to refusing sex, to disobeying her husband, to being unfaithful. In contrast, of women who have never experienced spousal violence, 45% of urban women and 73% of rural women agreed that violence was sometimes justified (8; Naripokkho. Findings from the household survey. 2006).

In other countries, strategies of women for coping with intimate partner or spousal violence generally use strong friendship networks (31). However, in Bangladesh, rates of disclosure of violence and help-seeking are very low. In a study of rural and urban women in 2001, two-thirds of women in violent relationships did not tell others about the violence they experienced or seek help (11). Immediate social networks (family, friends, and neighbours) rather than formal services provide the first point of contact for women in violent relationships (4,8,26). Only 2% of rural and urban women beaten by their husbands sought assistance from institutional sources, and only when the violence became life-threatening or their children were at risk. The generally low use of formal services reported reflects the limited availability of these services, difficulty in accessing these services, and perceptions that the service will not be able to help (11).

There are official channels of conflict resolution, but these are not yet well-understood or accessible to women who need to use them. The Government has established One-stop Crisis Centres in different districts in Bangladesh which facilitate person-centred counselling to abused women. A substantial barrier to institutionalizing counselling in all health-delivery points is the lack of trained professionals in this field, and this problem is more acute in rural areas as psychologists are reluctant to work in rural areas for extended periods. In an operations research context, ICDDR, B trained paramedics in spousal violence counselling. Preliminary findings from a study comparing the quality of counselling delivered by psychologists and paramedics in Matlab suggested that, with careful training and close supervision, primary-level healthcare professionals were able to provide some spousal violence counselling services (Naved RT *et al*. Personal communication, 2006).

Many factors inhibit staff in the healthcare system from reporting violence to the legal system. Healthcare system staff may lack the knowledge, time, and motivation to identify spousal violence and report it to the legal system. Staff members are poorly equipped to deal with the needs of battered or otherwise violated women. Greater awareness, professional competence, reliability, and strong support systems are necessary (26).

## DISCUSSION

The majority of women in Bangladesh have experienced spousal violence in their lifetimes. The consequences of such violence can be severe and include immediate injury and homicide, depression, poor health, and suicide. Young women, women with no education, and women just beginning income-generating activities have a higher risk of spousal violence than other women.

In its current state, the Bangladesh healthcare system is inadequately prepared to address spousal violence. Much exploratory and action research needs to be conducted to address the many unanswered questions about how to reduce spousal violence most effectively and efficiently in Bangladesh and eliminate it by 2015.

### Exploratory research

There is a need for more and better research on perpetrators of spousal violence and the proximate factors that may contribute to spousal violence. The list of possible proximate factors is long and inconclusive: husbands’ alcohol and drug use; husbands’ gambling; economic stress; differences in husbands’ and wives’ perceptions of women's and men's roles within marriage; differences in perceptions of the benefits of women's autonomy and income generation; and relational factors in the husband-wife dyad. These relational factors are an important subcategory and include: age at marriage; dowry agreements; difference in age of husband and wife and associated differences in expectations; arranged marriages and the reasoning associated with the arrangement (i.e. financial, for the happiness of the couple); expectations of sexual relations within marriage; and marital conflict and communication within marriage (6,11,26).

Research is required to explore the multifaceted consequences of violence for women, their children, their families, and communities. One of the consequences highlighted in this paper is suicide. Suicide is the number one cause of death among women of reproductive age in some age-groups in certain areas of Bangladesh. Suicide and associated factors need to be better understood as does the relationship between spousal violence and suicide. The very high and increasing rates of suicide among young unmarried women suggest that the relationships among premarital intimate partnerships, unwanted pregnancy, and suicide should be explored.

To facilitate the measurement trends of spousal violence and its correlates over time, spousal violence should be included in the existing public-health surveillance systems. One important lesson from this paper is that, without consistent methods applied to the same population over time, it is impossible to document the trends over time.

### Action research

Action research to develop and test interventions (preventive and curative) involving the Government of Bangladesh, health systems, women, men, communities, NGOs, and private-sector businesses is required to facilitate means of preventing spousal violence and of providing care, counselling, shelter, and rehabilitation for women who experience spousal violence and their children. For these activities to have a maximum impact, monitoring, evaluation, and sharing results efficiently are critical.

Programmes can address spousal violence on tertiary, secondary, and primary levels. Tertiary and secondary-level programmes aim at providing care and resources to women who have experienced spousal violence or are identified to be at risk of spousal violence. Primary prevention aims at behaviour change to prevent spousal violence (32).

Tertiary or secondary prevention measures can be developed and/or streamlined based on the information already available. Tertiary prevention measures include facility- or community-based screening of women with visible signs of abuse. Health systems are ideally positioned to screen such women for spousal violence, but to be effective on such a socially- and culturally-sensitive issue, these require staff competent in counselling, a working referral system, and active support of the social and legal systems. Systems need to be in place to demonstrate the benefits to woman of using such spousal violence support services. Because women often cite ‘shame to family’ as a barrier to seeking care for spousal violence, such programmes might demonstrate how they can have a positive impact on the family.

Bangladesh currently has a very few counsellors trained on issues around spousal violence. Operations research on a counselling initiative might include testing the impact of individual and group therapy counselling for victims.

Secondary prevention measures include identifying women most at risk of being exposed to spousal violence and providing them with the tools to seek help and support before they require assistance. Mechanisms for secondary prevention include facility- or community-based screening of women who may be most at risk—for example, young newly-married women, pregnant women, women with minimal or no formal education, and women who have recently joined the paid labour force.

Primary prevention to reduce spousal violence requires changing societal attitudes and behaviour to prevent spousal violence from occurring at all. One possible mechanism for primary prevention is universal screening of women. Such screening will facilitate the identification of women who have experienced spousal violence who may not otherwise have been identified and also will give the message to all women screened that spousal violence is an unacceptable behaviour, and a violation of women's rights for which women have recourse. Family-life education courses, including strategies for spousal communication and conflict resolution, particularly community-based, are important forums for spreading messages aiming at reducing spousal violence.

Primary prevention activities should also target men and boys who perpetrate or are at risk of perpetrating violence. Punitive measures against men who perpetrate spousal violence must be accompanied by rehabilitation and reintegration programmes. There are no systems in place in Bangladesh to help perpetrators of spousal violence develop or turn to strategies other than violence. Such systems need to be developed and carefully implemented and monitored.

### The health system's response to spousal violence against women

Unfortunately, there are minimal data on the training received by medical service providers in responding to persons who have been victims of spousal violence or the perpetrators of spousal violence. Few resources are available from the health systems to provide counselling or otherwise help women and their husbands to address the abuse. Much research is needed to understand how to help clinicians provide proper services to these patients, and how the health and legal systems should cooperate effectively and efficiently to reduce the incidence of spousal violence.

### Summary

The issue of spousal violence against women is complex and is culturally and socially sensitive. Nonetheless, the Government has demonstrated its commitment to eliminating violence against women in a short time. Research institutions, non-governmental and civil society organizations have critical roles to play in working with the Government of Bangladesh as a united front to reduce violence against women and the associated and unnecessary physical and mental anguish, death, and disability.
